# Elementary Flux Modes Analysis of Functional Domain Networks Allows a Better Metabolic Pathway Interpretation

**DOI:** 10.1371/journal.pone.0076143

**Published:** 2013-10-29

**Authors:** Sabine Pérès, Liza Felicori, Franck Molina

**Affiliations:** 1 Laboratoire de Recherche en Informatique, Université Paris-Sud, CNRS UMR 8623 and INRIA Saclay, Orsay, France; 2 Universidade Federal de Minas Gerais, Bioquimica e Imunologia, Belo Horizonte, Brazil; 3 SysDiag UMR3145 CNRS/Bio-Rad Parc Euromédecine, Montpellier, France; Semmelweis University, Hungary

## Abstract

Metabolic network analysis is an important step for the functional understanding of biological systems. In these networks, enzymes are made of one or more functional domains often involved in different catalytic activities. Elementary flux mode (EFM) analysis is a method of choice for the topological studies of these enzymatic networks. In this article, we propose to use an EFM approach on networks that encompass available knowledge on structure-function. We introduce a new method that allows to represent the metabolic networks as functional domain networks and provides an application of the algorithm for computing elementary flux modes to analyse them. Any EFM that can be represented using the classical representation can be represented using our functional domain network representation but the fine-grained feature of functional domain networks allows to highlight new connections in EFMs. This methodology is applied to the tricarboxylic acid cycle (TCA cycle) of *Bacillus subtilis*, and compared to the classical analyses. This new method of analysis of the functional domain network reveals that a specific inhibition on the second domain of the lipoamide dehydrogenase (pdhD) component of pyruvate dehydrogenase complex leads to the loss of all fluxes. Such conclusion was not predictable in the classical approach.

## Introduction

Metabolic pathway analysis is important for assessing network properties in (reconstructed) biochemical reaction networks [1], [2]. Numerous biotechnological applications of this kind of analysis exist, mainly in the metabolic engineering field. This includes the extension of existing pathways to achieve the synthesis of novel products, to redirect metabolite fluxes towards a desired product, and to accelerate or bypass steps that exert high flux control [3].

The notion of elementary flux mode (EFM) is a key concept derived from the analysis of metabolic networks from a pathway-oriented perspective. An EFM is defined as the smallest sub-network that enables the metabolic system to operate at steady state with all irreversible reactions proceeding in the appropriate direction [4], [5]. In metabolic pathway analyses, the metabolic networks are described in terms of biochemical reactions, enzymes and metabolites. However, it was previously shown that enzymes (proteins) functions are a composition of elementary actions supported by submolecular functional units: the protein domains [6], [7]. In nature, proteins are mainly made of more than one domain. In previous work, Zhang [8] revealed that proteins forming the network of the bacterium *Thermotoga maritima* are dominated by a small number of structural domains performing diverse but mostly related functions. This new type of description provides insight into the evolution of metabolic networks [9]. Consequently, the identification of functional protein domains supported by specific folds in networks can therefore provide insights into their function and maybe change the paradigm of network analysis.

An essential aspect of our work is to represent the metabolic networks using a new paradigm by looking at the enzymes functional units based on the protein domains instead of enzymes represented as a whole entity. Protein domains are part of the protein showing specific sub-functions as functional units (and often sub 3D structure). Most of the proteins are made of different domains. For a given metabolism, such a modification in enzymatic activity representation leads to a metabolic network made of functional units (domain with a new set of connectivities) but representing the same metabolism. We based our approach on this newly represented functional networks and we calculated the elementary flux modes to check whether the EFMs of the classical representation can be represented using the functional domain network and to identify new ones. We illustrated our approach with the tricarboxylic acid cycle (TCA cycle) of the bacteria *Bacillus subtilis*. We observed that enzymes-domains EFM analysis, in the case of the TCA cycle, has revealed the key role of the domain involved in NAD/FAD binding. This kind of observation has several implications in the domain of systems and synthetic biology and could help biologists to test, not only the role of the enzymes, but the domains in biological systems and to create new pathways and biological functions.

## Methods

### Domain Function Assignment

To build a metabolic network of functional units represented by enzyme domains, we have to identify for each enzyme, its structural domains and the elementary actions they provide. To identify the structural domains we perform a systematic molecular modelling of all enzymes of the network, thanks to a pipeline dedicated to protein structure modelling using Python and Perl routines, and use the homology modelling software Modeller version 9v4 [10], [11].

Briefly said, this routine recursively takes the list of target sequences as input file (profile.py) and does a multiple (multalign.pl) or single alignment (salign.py), then generates the model (model.py). The best model of each enzyme is selected based on the lowest modeller objective function score (MOF). After this step, the structural domains of the proteins are assigned to each of the protein models using fastSCOP [12], [13]. Besides, fastSCOP, PFAM [14], [15], Swiss-Prot [16] and literature review were used for the functional domain assignment.

**Figure 1:**
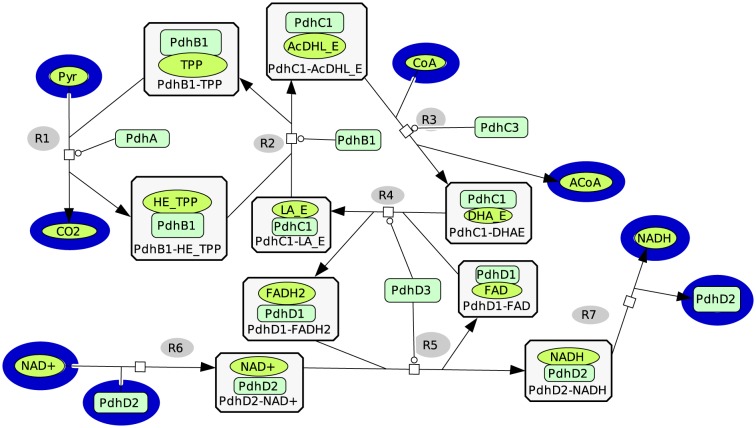
Functional domains network of the pyruvate dehydrogenase complex. The network was designed using CellDesigner [32] with SBGN [33] (see Table 5 for the abbreviations). The rectangular nodes represent the enzymes' domains and the oval nodes represent the metabolites. The external nodes are surrounded by a blue outline. The symbol 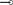 represents the catalytic activities on the reactions.

We illustrate our approach on the *Bacillus subtilis* pyruvate dehydrogenase multienzyme complex (PDH). This complex, encoded by the pdhABCD operon, is composed of three different enzymes: enzyme 1 or pyruvate decarboxylase, composed of alpha and beta subunits, coded by pdhA and pdhB respectively; enzyme 2 or dihydrolipoyl transacetylase coded by pdhC, and enzyme 3 or lipoamide dehydrogenase coded by pdhD. After the molecular modelling and the structural and functional domain assignment, we can observe that these enzymes are composed of 1 to 3 domains, each of them responsible for different activities (Table 1) composing the overal protein function. For instance, the domains c.36.1 from alpha (pdhA) and beta-subunits (pdhB) of enzyme 1 bind thiamine pyrophosphate (TPP) in both cases. However, while the c.36.1 domain from pdhA catalyses pyruvate decarboxylation, the c.36.1 domain from pdhB catalyses the second reaction of the acetyl group with the lipoate, as described in *Geobacillus stearothermophilus* [17], see (Table 1, Figure 1).

**Table 1:** Pyruvate dehydrogenase complex enzyme decomposition in structural domain according to scop fold classification.

Gene name/enzyme	structural domain	domain function	label in the
	(scop)		Fig. 1 & 2 & 3
PdhA/enzyme 1	c.36.1	Binding TPP and active site (first reaction)	PdhA
PdhB/enzyme 1	c.36.1	Binding TPP and active site (second reaction)	PdhB1
	c.48.1	regulatory binding site	PdhB2
PdhC/enzyme 2	b.84.1	Lipoyl binding	PdhC1
	a.9.1	E1/E3 binding	PdhC2
	c.43.1	Active site	PdhC3
PdhD/enzyme 3	c.3.1	Binding FAD	PdhD1
	c.4.1	Binding NAD/FAD	PdhD2
	d.87.1	Active site	PdhD3

### Functional Domain Network Design and Analysis

Another objective of this work was to represent the metabolic networks in a different way, by looking at the enzymes' functionalities supported by the enzyme domains instead of the enzyme as a whole entity. As soon as the activities of each domain are obtained, the functional domain networks can be built. A functional domain network is now defined as a set of biological activities which can be involved in enzymatic reactions or molecular interactions. It is represented by an oriented hypergraph where the nodes represent the molecular objects (metabolites, enzyme domains) and the arrows represent the reactions or the molecular interactions. The symbol 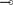 represents the catalytic activities on the reactions. The domains having a binding activity are represented by nodes which connect the arrows (*i.e* the reactions) and the domains having an enzymatic activity are represented by nodes which are linked to the reaction with the symbol 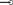.

Our representation of metabolic networks as functional domain networks is just a step forward to implement details on structure/function of molecules involved in a given metabolic networks. The change of representation method does not modify the role of the network to metabolize biochemical compounds (small molecule). By implementing structural (3D domain organization) and functional details, our representation has to deal with elementary actions either binding-related (interaction) or chemical transformation-related (catalytic). We decided to analyze such a new network representation with EFMs since it has already been proved that EFMs can deal with both interaction and chemical transformations [18].

Based on this formalism, we have built the new pyruvate dehydrogenase functional domain network (see Figure 1). The functional domain network representation, compared to the classical one, allows an enriched description of the functional paths of the network. To evaluate this detailed representation, we calculated the elementary flux modes with metatool [19], [20]. Elementary flux mode analysis is a well-known method to exhibit all the possible pathways within a given network. For the functional domain network representing the PDH complex, we obtained one elementary flux mode which contains all the reactions. The metatool input and the sbml files can be found in File S1. Compared to the classical representation, as expected, this single elementary flux mode has the same overall reaction: Pyruvate+NAD^+^+CoA→Acetyl-CoA+CO_2_+NADH. This short example shows that the functional domain network representation is functionally consistent with the classical representation. Interestingly, it contains much more functional details which can be later used for further in depth interpretations in complex systems.

## Results

### Traditional description of the TCA cycle

**Figure 2:**
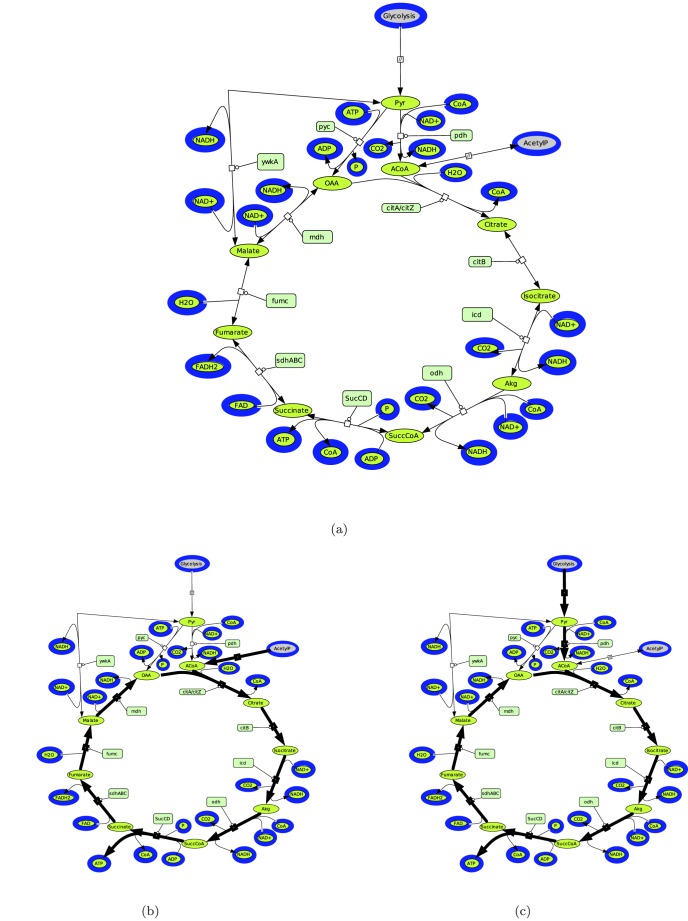
Classical description of TCA cycle of *Bacillus Subtilis* and the two elementary flux modes which produce ATP. (A) Classical description of TCA cycle of *Bacillus Subtilis* (see table 5 for the abbreviations). (B–C) EFMs which produce ATP. The reactions belonging to the EFMs are in bold. (B) ATP production with an input of Acetyl-phosphate. (C) ATP production with an input coming from the glycolysis.

The citric or tricarboxylic acid cycle (TCA cycle) is the metabolic pathway that oxidizes acetyl-CoA to carbon dioxide [21] and plays a central role in the metabolism of *Bacillus* species and most other cells (see Figure 2(a)). Recently, we showed the importance of the nature of the carbon source in enzymes expression and metabolic quantification of TCA cycle components [22]. Although this metabolic network is well-known, our new approach reveals a reorganisation of fluxes in the network. This reorganisation of fluxes is caused by the finer description of molecular funtional units which in turn induces a rearrangement of the connectivities in the hypergraph. This seems to be frequent in networks. To anticipate such a hidden complexity, one needs a more detailed fluxes description of networks, as the one offered by the functional domain network description. We computed elementary flux modes using metatool [19], [20] on the classical description of TCA network. The metatool input and the sbml files can be found in File S2. There are six elementary flux modes (see Table 2), only two out of them, EFM 2 and EFM 5, produce ATP. They are represented in the Figure 2(b) and Figure 2(c).

**Table 2:** Elementary flux modes of TCA cycle and their overall reactions.

EFM 1	pyc -mdh ywka
Overall reaction 1	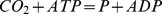
EFM 2	citA odh citB icd -SucCD SucABC fumC mdh -Pta
Overall reaction 2	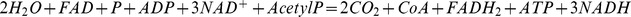
EFM 3	citA pyc odh citB icd -SucCD SucABC fumC ywka -Pta
Overall reaction 3	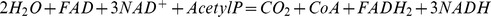
EFM 4	pdh Glyco Pta
Overall reaction 4	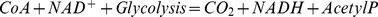
EFM 5	pdh citA odh Glyco citB icd -SucCD SucABC fumC mdh
Overall reaction 5	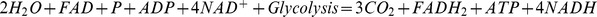
EFM 6	pdh citA pyc odh Glyco citB icd -SucCD SucABC fumC ywka
Overall reaction 6	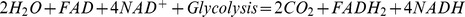

We can note that if the PDH is inhibited then the EFM 2 is not functional. It is however still possible to produce ATP through the EFM 1. The functional domains representation presented in the previous section, shows that in fact, the PDH is the result of several enzymatic activities involving reaction intermediates. The fluxes extracted from the classical TCA network description do not explicitly take into account these intermediate reactions where enzymatic domains and intermediate metabolites are involved.

### TCA cycle network using domains as enzymatic units

**Figure 3:**
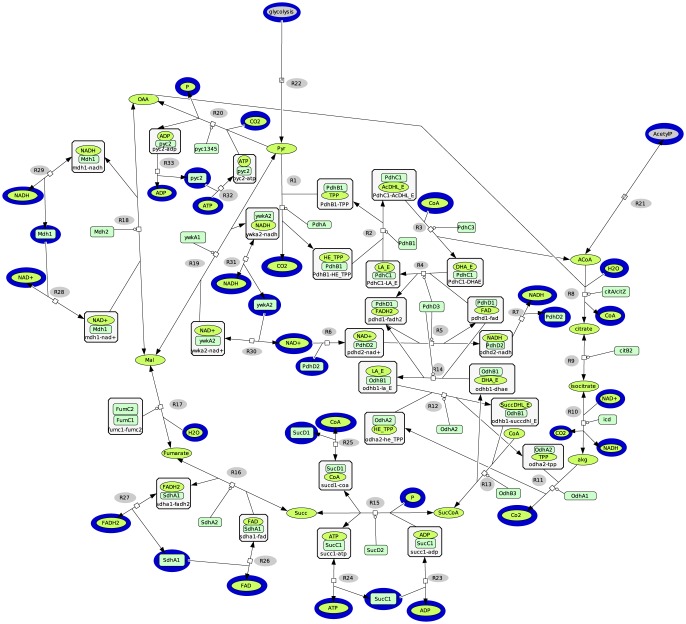
Functional domains network of the TCA cycle. The rectangular nodes represent the enzymes' domains and the oval nodes represent the metabolites. The external nodes are surrounded in a blue compartment. The symbol 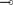 represents the catalytic activities on the reactions. The functional domains network contains the PDH (R1–R7), the citrate synthase (R8), the aconitase (R9), the isocitrate dehydrogenase (R10), the *α*–ketoglutarate dehydrogenase (R11–R14, R5–R7), the succinyl-CoA synthetase (R15), the succinate dehydrogenase (R16), the fumarase (R17), the malate dehydrogenase (R18), the malic enzyme (R19), the pyruvate carboxylase (R20), the input of Acetyl-CoA from AcetylP (R21), the input of pyruvate from the glycolysis (R22), the binding of ATP/ADP with SucC1 (R23–R24), the binding of CoA with SucD1 (R25), the binding of FAD/FADH2 with SdhA1 (R26–R27), the binding of NAD+/NADH with Mdh1 (R28–R29), the binding of NAD+/NADH with ywkA2 (R30–R31), the binding of ATP/ADP with pyc2 (R32–R33).

To enrich the metabolic network description of the TCA cycle with functional enzyme domains, we applied the same methodology as the one used to describe pyruvate dehydrogenase complex in the previous section. To perform domain identification and functional assignment, we used all the sequences of all TCA enzymes from *subtilist* database [23] and the methodology presented above for the 3D modelling and domain assignments. Our functional domains description allows to emphasize that the PDH complex and the *α*–ketoglutarate dehydrogenase (ODH) complex have similar mechanisms and similar domains structures. Moreover, Hoch and Coukoulis [24] shown that *α*–ketoglutarate dehydrogenase contains the same subunit (pdhD) as the PDH complex of *Bacillus subtilis*. Based on the activities of each domain, see Table 3, we reconstructed the TCA cycle (see Figure 3) and calculated the EFMs. The metatool input and the sbml files can be found in File S3.

**Table 3:** Decomposition of TCA enzymes in structural domain according to scop fold classification.

Gene name/enzyme	structural domain	domain function	label in the
	(scop)		Fig. 3
citA/citrate synthase	a.103.1	Active site	citA
citZ/citrate synthase	a.103.1	Active site	citZ
citB/aconitase	c.83.1	Binding Iron-sulfur	
	c.8.2	Active site	citB2
icd/isocitrate DH	c.77.1	Active site	icd
odhA/*α*–ketoglutarate A	c.36.1	Binding TPP and Active site	OdhA1
	c.36.1	Binding TPP	OdhA2
	c.48.1	Active site	OdhA3
odhB/*α*–ketoglutarate B	b.84.1	Lipoyl binding	OdhB1
	a.9.1	pdhD binding	OdhB2
	c.43.1	Active site	OdhB3
PdhD/enzyme 3	c.3.1	Binding FAD	PdhD1
	c.4.1	Binding NAD/FAD	PdhD2
	d.87.1	Active site	PdhD3
SucC/Succinyl-CoA synthetase	d.142.1	ATP graps	SucC1
	c.23.4	CoA Ligase	SucC2
SucD/Succinyl-CoA synthetase	c.2.1	CoA binding	SucD1
	c.23.4	active site	SucD2
sdhA/succinate dehydrogenase	c.3.1	FAD binding	sdhA1
	d.168.1	active site	sdhA2
	c.3.1	FAD/NAD(P)-binding domain	sdhA3
	a.7.3	Succinate dehydrogenase/	sdhA4
		fumarate reductase flavoprotein C-terminal domain	
sdhB/succinate dehydrogenase	d.15.4	2Fe-2S ferredoxin-like	sdhB1
	a.1.2	alpha-helical ferredoxin	sdhB2
sdhC/succinate dehydrogenase	f.21.2	Fumarate reductase respiratory complex	sdhC
		transmembrane subunits	
fumC/fumarase	a.127.1	L-aspartase-like	fumC1
	a.127.1		fumC2
mdh/malate dehydrogenase	c.2.1	binding site	mdh1
	d.162.1	active site	mdh2
ywkA/malic enzyme	c.58.1	active site	ywkA1
	c.2.1	NAD binding	ywkA2
pyc/pyruvate carboxylase	c.30.1	PreATP-grasp domain	pyc1
	d.142.1	Glutathione synthetase ATP-binding domain-like	pyc2
	b.84.2	Rudiment single hybrid motif	pyc3
	c.1.10	Aldolase	pyc4
	a.5.7	post-HMGL domain-like	pyc5

**Figure 4:**
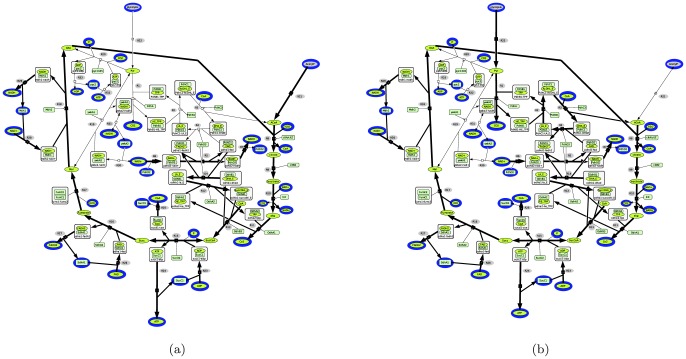
Elementary flux modes which produce ATP of the functional domains network of the TCA cycle. (A) ATP production with an input of Acetyl-phosphate. (B) ATP production with an input coming from the glycolysis.

In this system, there are again six elementary flux modes performing the same overall reactions but they are described in a finer way compared to the classical TCA network representation (Table 4). Some of them additionally contain the domain SucD1 because it is now considered as an external node. Enzymatic activities supported by domains can be affected by their local environment independently from the rest of the molecule (inhibition, activation, kinetics, etc.). Figure 4 shows the two out of the six domain-based elementary flux modes (EFM 2 and EFM 5) which produce ATP. They involve the PDH complex and the ODH complex. The reactions which are linked to PdhD2 (R5, R6, R7) are involved in five EFMs including the two producing ATP. If, for instance, the R5 activity is inhibited by a compound from the local environment, this could lead to suppression of the five EFMs. Then, none of the EFMs of the system could produce ATP anymore. It is thus worth noticing that using the functional domain network changes the topology of the hypergraph in adding connectivities. Hence, it links an input branch (the PDH complex) with the cycle by the lipoamide dehydrogenase (pdhD). Thus, influencing a domain of the lipoamide dehydrogenase suddendly affects the EFMs of the network even though they were not affected in the classical representation.

**Table 4:** Elementary flux modes of functional domain network of TCA cycle and their overall reactions.

EFM 1	R20 -R18 R19 -R28 -R29 R30 -R31 R32 R33
Overall reaction 1	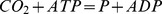
EFM 2	R5 R6 R7 R8 R11 R12 R13 R14 R9 R10 R15 R16 R17 R18 -R21 R23 R24 -R25 R26 R27 R28 R29
Overall reaction 2	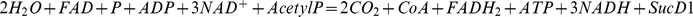
EFM 3	R5 R6 R7 R8 R11 R12 R13 R14 R20 R9 R10 R15 R16 R17 R19 -R21 R23 R24 -R25 R26
	R27 R30 -R31 R32 R33
Overall reaction 3	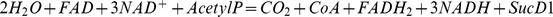
EFM 4	R1 R2 R3 R4 R5 R6 R7 R22 R21
Overall reaction 4	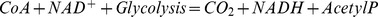
EFM 5	R1 R2 R3 R4 (2 R5) (2 R6) (2 R7) R8 R11 R12 R13 R14 R22 R9 R10 R15 R16 R17 R18 R23
	R24 -R25 R26 R27 R28 R29
Overall reaction 5	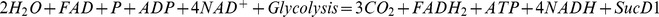
EFM 6	R1 R2 R3 R4 (2 R5) (2 R6) (2 R7) R8 R11 R12 R13 R14 R20 R22 R9 R10 R15 R16 R17 R19
	R23 R24 -R25 R26 R27 R30 -R31 R32 R33
Overall reaction 6	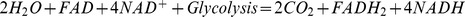

**Table 5:** Abbreviations of metabolites.

Pyr	pyruvate
CoA	coenzyme A
ACoA	acetyl-CoA
AKG	*α*-ketoglutarate
SucCoA	succinyl-CoA
OAA	oxaloacetate
AcCoA	acetyl-CoA
NAD/NADH	nicotiamide adenine dinucleotide (coenzyme red-ox)
FAD/FADH2	flavine adenine dinucleotide (coenzyme red-ox)
ATP	adenosine triphosphate
ADP	adenosine diphosphate
TPP	thiamine diphosphate
HE-TPP	2-hydroxyethyl-TPP
LA_E	lipoamide_E
AcDHL_E	acetyl-dihydrolipoamide_E
DHA_E	dihydrolipoamide_E
SucDHL_E	succinyl-dihydrolipoamide_E

On the other hand, if an intermediate metabolite described in our new representation is consumed by an enzymatic activity in the local environment, then our functional domain representation allows to show it. This was not predictable in the classical system. Because our functional domain network description is more detailed, it allows to address and analyse in a finer way the metabolic reactions in a network and predict the crucial role of the specific NAD/FAD domain (c.4.1) of lipoamide dehydrogenase (pdhD) in ATP production.

## Discussion

The understanding and engineering of metabolic networks requires powerful theoretical methods such as pathway analysis, in which the topology of metabolic networks is considered. Previous works already showed the interest in looking at the detailed sub-molecular organization in metabolic networks [8]. However such a detailed molecular function description has never been used for global metabolic network analyses. Moreover, even if some efforts have been done to identify and annotate protein domains [25]–[28] and to predict domain-domain interactions [29], they did not used EFMs description to analyze functional domain networks. In this work we have provided a powerful method for network analysis, using not only the classical description of EFMs, but also a detailed analysis of enzyme domains. This methodology combines structural domain and functional analysis of enzymes and produces a new functional domains network representation which allows a finer description of successive reactions. This detailed representation is suitable for EFM analysis. We showed that the functional domain network EFM analysis provides a detailed description of the same overall reaction. When this approach was applied on the TCA cycle of *Bacillus subtilis* we showed that the analysis of EFMs of domain networks can lead to additional conclusions compared to the analysis of EFMs from classical metabolic networks. In particular, it is now possible to take into account interactions with the local environment of newly described intermediate reactions and metabolites. In our analysis of the TCA cycle, we observed that the enzymes analysed are made of 1 to 5 domains that perform different roles in the system. This detailed knowledge about enzyme domains functions was used in the EFM analysis. Our results showed that we gain detailed informations about the key role of a given domain in a functional domain network. It is the case of the second domain of enzyme PdhD, also found in two different enzymes, and responsible for NAD/FAD binding. Lipoamide dehydrogenase (pdhD) or Lpd, the third enzyme in *Mycobacterium tuberculosis*'s pyruvate dehydrogenase complex (PDH), helps *Mycobacterium tuberculosis* to resist host reactive nitrogen intermediates. Recent works on this specific enzyme complex show that Lpd is a potential target for anti-infectives against *Mycobacterium tuberculosis*. The authors clearly identify the need of a finer functional network description, to drive their drug-design strategy as complement to gene knockout studies [30].

One limitation of this kind of analysis is the availability of detailed informations about enzyme activities and the annotation of their domain-supported functions. This requires strong interaction between structural and functional enzymatic activities. Our method, combining the representation of metabolic networks at the functional molecular domain level and the EFM analysis, allows more relevant functional studies. In the context of synthetic biology where biological networks are engineered, our method can provide a robust way to perform relevant functional analysis. This should strongly impact our capacity to better anticipate the potential behaviour of a newly designed synthetic network [31]. When used on natural biological networks, our method can also help to discover hidden non-obvious pathways.

## Supporting Information

File S1 **Metatool input and the sbml files of the PDH domain model.**
(ZIP)

File S2 **Metatool input and the sbml files of the classical TCA model.**
(ZIP)

File S3 **Metatool input and the sbml files of the TCA domain model.**
(ZIP)

## References

[1] Ruppin E, Papin JA, de Figueiredo LF, Schuster S (2010) Metabolic reconstruction, constraint- based analysis and game theory to probe genome-scale metabolic networks. Current Opinion in Biotechnology 21: 502–510. 20692823 10.1016/j.copbio.2010.07.002

[2] Ro D, Paradise E, Ouellet M, Fisher K, Newman K, et al (2006) Production of the antimalarial drug precursor artemisinic acid in engineered yeast. Nature 440: 940–943. 16612385 10.1038/nature04640

[3] Schuster S, Dandekar T, Fell D (1999) Detection of elementary modes in biochemical networks : A promising tool for pathway analysis and metabolic engineering. Trends Biotechnol 17: 53–60. 10087604 10.1016/s0167-7799(98)01290-6

[4] Schuster S, Hilgetag C (1994) On elementary flux modes in biochemical reaction systems at steady state. Journal of Biological Systems 2: 165–182.

[5] Schuster S, Fell D, Dandekar T (2000) A general definition of metabolic pathways useful for systematic organization and analysis of complex metabolic networks. Nat Biotechnol 18: 326–332. 10700151 10.1038/73786

[6] Peres S, Felicori L, Rialle S, Jobard E, Molina F (2010) Computing biological functions using bio*ψ*, a formal description of biological processes based on elementary bricks of actions. Bioinformatics 26: 1542–1547. 20448138 10.1093/bioinformatics/btq169 PMC2881354

[7] Mazière P, Granier C, Molina F (2004) A biological processes description scheme based on elementary bricks of action. J Mol Biol 339: 77–88. 15123422 10.1016/j.jmb.2004.03.029

[8] Zhang Y, Thiele I, Weekes D, Li Z, Jaroszewski L, et al (2009) Three-dimensional structural view of the central metabolic network of thermotoga maritima. Science 325: 1544–1549. 19762644 10.1126/science.1174671 PMC2833182

[9] Betts MJ, Russell RB (2009) A more structured metabolome. Nature Structural & Molecular Biology 16: 1125–1126. 10.1038/nsmb1109-1125 19888310

[10] A Sali TB (1993) Comparative protein modelling by satisfaction of spatial restraints. J Mol Biol 234: 779–815. 8254673 10.1006/jmbi.1993.1626

[11] Eswar N, Webb B, Marti-Renom MA, Madhusudhan M, Eramian D, et al.. (2002) Comparative Protein Structure Modeling Using Modeller, John Wiley & Sons, Inc. doi:10.1002/0471250953. bi0506s15. Available: http://dx.doi.org/10.1002/0471250953.bi0506s15.

[12] Tung C, Yang J (2007) fastscop: a fast web server for recognizing protein structural domains and scop superfamilies. Nucleic Acids Research 35: 438–443. 10.1093/nar/gkm288 PMC1933144 17485476

[13] Fastscop website. Available: http://fastscop.life.nctu.edu.tw. Accessed 2013 may 24.

[14] Finn RD, Mistry J, Tate J, Coggill P, Heger A, et al (2010) The Pfam protein families database. Nucleic Acids Research 38: 211–222. 10.1093/nar/gkp985 PMC2808889 19920124

[15] Pfam website. Available: http://pfam.sanger.ac.uk. Accessed 2013 may 24.

[16] Swissprot website. Available: http://www.expasy.ch/sprot. Accessed 2013 may 24.

[17] Pei X, Titman C, Frank R, Leeper F, Luisi B (2008) Snapshots of catalysis in the e1 subunit of the pyruvate dehydrogenase multienzyme complex. Structure 16: 1860–72. 19081062 10.1016/j.str.2008.10.009 PMC2663715

[18] Klamt S, Saez-Rodriguez J, Lindquist JA, Simeoni L, Gilles ED (2006) A methodology for the structural and functional analysis of signaling and regulatory networks. BMC Bioinformatics 7 10.1186/1471-2105-7-56 PMC1458363 16464248

[19] Pfeiffer T, Sanchez-Valdenebro I, Nuno J, Montero F, Schuster S (1999) Metatool: for studying metabolic networks. Bioinformatics 15: 251–257. 10222413 10.1093/bioinformatics/15.3.251

[20] Von Kamp A, Schuster S (2006) Metatool 5.0: Fast and exible elementary modes analysis. Bioinformatics 22: 1930–1931. 16731697 10.1093/bioinformatics/btl267

[21] Sonenshein AL (2002) The krebs citric acid cycle. Bacillus subtilis and its closest relatives: from genes to cells ASM Press, Washington, DC 151–162.

[22] Buescher JM, Liebermeister W, Jules M, Uhr M, Muntel J, et al (2012) Global network reorganization during dynamic adaptations of Bacillus subtilis metabolism. Science 335: 1099–103. 22383848 10.1126/science.1206871

[23] Subtilist website. Available: http://genolist.pasteur.fr/subtilist. Accessed 2013 may 24.

[24] Hoch J, Coukoulis H (1978) Genetics of the alpha-ketoglutarate dehydrogenase complex of bacillus subtilis. J Bacteriol 133: 265–9. 412834 10.1128/jb.133.1.265-269.1978 PMC222003

[25] Andreeva A, Howorth D, Chandonia JM, Brenner SE, Hubbard TJP, et al (2008) Data growth and its impact on the scop database: new developments. Nucleic Acids Research 36: D419–D425. 18000004 10.1093/nar/gkm993 PMC2238974

[26] Letunic I, Doerks T, Bork P (2009) Smart 6: recent updates and new developments. Nucleic Acids Research 37: 229–232. 10.1093/nar/gkn808 PMC2686533 18978020

[27] Letunic I, Doerks T, Bork P (2012) Smart 7: recent updates to the protein domain annotation resource. Nucleic Acids Research 40: 302–305. 10.1093/nar/gkr931 PMC3245027 22053084

[28] Cuff AL, Sillitoe I, Lewis T, Clegg AB, Rentzsch R, et al (2011) Extending cath: increasing coverage of the protein structure universe and linking structure with function. Nucleic Acids Research 39: D420–D426. 21097779 10.1093/nar/gkq1001 PMC3013636

[29] Yellaboina S, Tasneem A, Zaykin DV, Raghavachari B, Jothi R (2011) Domine: a comprehensive collection of known and predicted domain-domain interactions. Nucleic Acids Research 39: 730–735. 10.1093/nar/gkq1229 PMC3013741 21113022

[30] Venugopal A, Bryk R, Shi S, Rhee K, Rath P, et al (2011) Virulence of mycobacterium tuberculosis depends on lipoamide dehydrogenase, a member of three multienzyme complexes. Cell host & microbe 9: 21–31. 21238944 10.1016/j.chom.2010.12.004 PMC3040420

[31] Rialle S, Felicori L, Dias-Lopes C, Peres S, El Atia S, et al (2010) Bionetcad: design, simulation and experimental validation of synthetic biochemical networks. Bioinformatics 26: 2298–2304. 20628073 10.1093/bioinformatics/btq409 PMC2935418

[32] Funahashi A, Matsuoka Y, Jouraku A, Morohashi M, Kikuchi N, et al (2008) Celldesigner 3.5: A versatile modeling tool for biochemical networks. Proceedings of the IEEE 96: 1254–1265.

[33] Le Novère N, Hucka M, Mi H, Moodie S, Schreiber F, et al (2009) The systems biology graphical notation. Nat Biotechnol 8: 735–741. 10.1038/nbt.1558 19668183

